# Defective Sphingosine-1-phosphate metabolism is a druggable target in Huntington’s disease

**DOI:** 10.1038/s41598-017-05709-y

**Published:** 2017-07-13

**Authors:** Alba Di Pardo, Enrico Amico, Abdul Basit, Andrea Armirotti, Piyush Joshi, M. Diana Neely, Romina Vuono, Salvatore Castaldo, Anna F. Digilio, Francesco Scalabrì, Giuseppe Pepe, Francesca Elifani, Michele Madonna, Se Kyoo Jeong, Bu-Mahn Park, Maurizio D’Esposito, Aaron B. Bowman, Roger A. Barker, Vittorio Maglione

**Affiliations:** 1 IRCCS Neuromed, Pozzilli, Italy; 2 Department of Drug Discovery and Development, Fondazione Istituto Italiano di Tecnologia, Genova, Italy; 3 Departments of Pediatrics, Neurology and Biochemistry, Vanderbilt University (VU) and VU Medical Center Pediatric Neurology Research Lab, Nashville, TN USA; 4 John van Geest Cambridge Centre for Brain Repair, Department of Clinical Neuroscience, University of Cambridge, Cambridge, UK; 5 Institute of Biosciences and Bioresources (IBBR), National Research Council (CNR), Naples, Italy; 6 Department of of Cosmetic Science, Seowon University, Cheongju, Korea; 7 NeoPharm USA Inc. Engelwood Cliffs, New Jersey, USA; 8 Institute of Genetics and Biophysics “A. Buzzati-Traverso”, Naples, Italy

## Abstract

Huntington’s disease is characterized by a complex and heterogeneous pathogenic profile. Studies have shown that disturbance in lipid homeostasis may represent a critical determinant in the progression of several neurodegenerative disorders. The recognition of perturbed lipid metabolism is only recently becoming evident in HD. In order to provide more insight into the nature of such a perturbation and into the effect its modulation may have in HD pathology, we investigated the metabolism of Sphingosine-1-phosphate (S1P), one of the most important bioactive lipids, in both animal models and patient samples. Here, we demonstrated that S1P metabolism is significantly disrupted in HD even at early stage of the disease and importantly, we revealed that such a dysfunction represents a common denominator among multiple disease models ranging from cells to humans through mouse models. Interestingly, the *in vitro* anti-apoptotic and the pro-survival actions seen after modulation of S1P-metabolizing enzymes allows this axis to emerge as a new druggable target and unfolds its promising therapeutic potential for the development of more effective and targeted interventions against this incurable condition.

## Introduction

Huntington’s disease (HD), the most common dominantly inherited neurodegenerative disorder affecting an estimated 3 to 7 per 100,000 people, is associated with progressive motor, cognitive and behavioral disturbances^1^. The disease-causing mutation is an expansion of CAG trinucleotide repeats (>36 repeats) within the *HTT* gene encoding a polyglutamine (polyQ) stretch in the N-terminal region of Huntingtin (Htt), a ubiquitous protein whose function is still unclear^2^. Expansion of the polyQ stretch endows mutant Htt (mHtt) with toxic properties and results in a range of cellular abnormalities^3^ including aberrant metabolism of brain lipids^4–9^.

**Figure 1:**
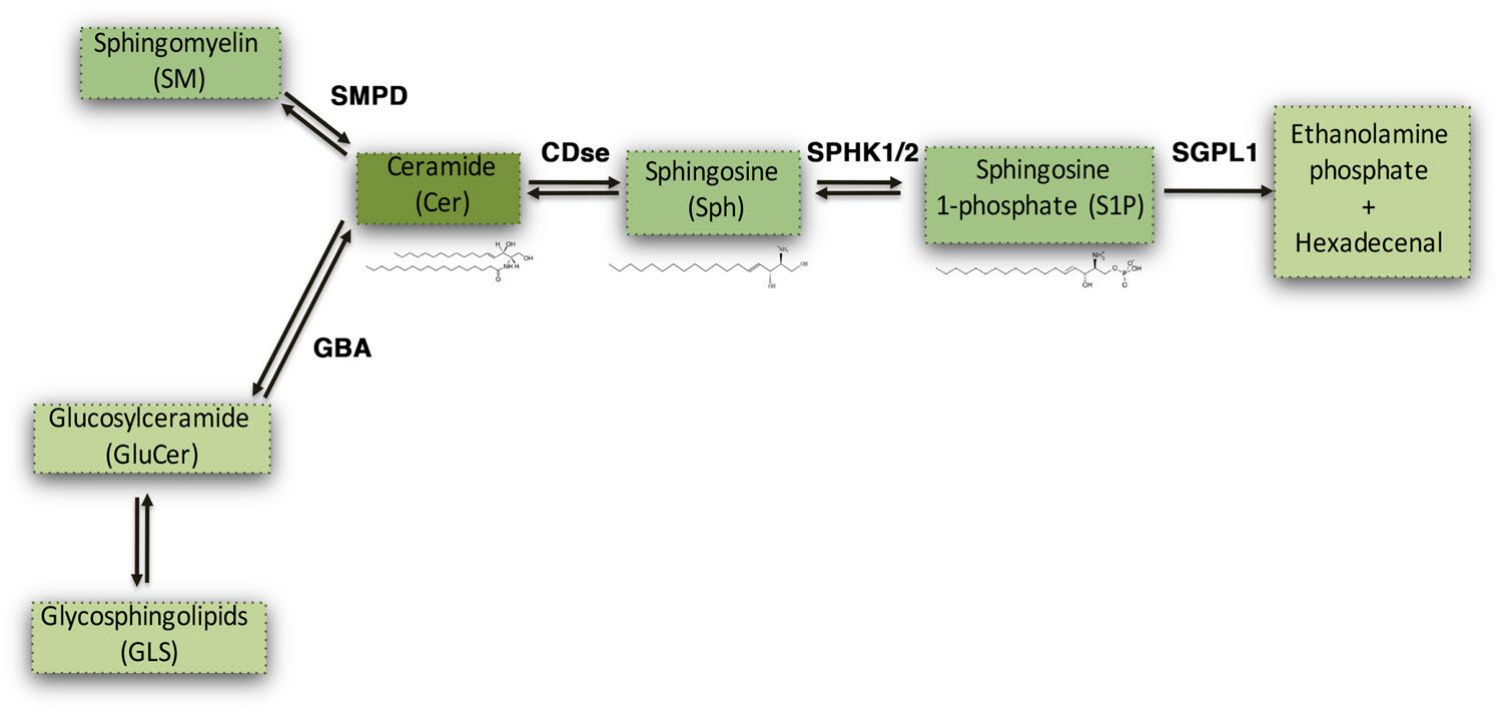
Simplified sphingolipid metabolic pathway. Ceramide (Cer) is generated through the degradation of either Sphingomyelin (SM) or Glycosphingolipids(GLS) by Sphingomyelin Phosphodiesterase (SMPD) and GlucosylCeramidase (GBA) respectively. Cer is subsequently metabolized by Ceramidase (CDse) to generate Sphingosine (Sph), which in turn produces Sphingosine-1-Phosphate (S1P) through phosphorylation by Sphingosine Kinase-1 and Sphingosine Kinase-2 (SPHK1/2). All these reactions are reversible. S1P can be catabolized into hexadecenal and  phospho-ethanolamine by S1P Lyase (SGPL1).

Sphingolipids represent the major lipid component of biological membranes and regulate a number of important cellular functions^10^. Ceramide (Cer), sphingosine (Sph) and its phosphorylated form Sphingosine-1-phosphate (S1P) are key intermediates in the sphingolipid metabolism^10,11^ (Fig. 1) and thus tightly regulated. S1P is found both within the intracellular and extracellular compartments^12–14^ and outside the cell, it acts as a high affinity agonist at five known G protein-coupled receptors, which are highly expressed in the brain^12,15^.

S1P metabolism is a complex process and normally implicates a number of different enzymes (see Fig. 1). Sphingosine kinase 1 and 2 (SPHK1/2) catalyze the phosphorylation of Sph to generate S1P^16^. Under normal conditions, SPHK1 activity is associated with cell survival^17,18^, while SPHK2 has a more complex effect – coordinating a number of intracellular pathways such that it leads to loss of cell growth and apoptosis when altered^18–22^. On the other hand, the degradative enzyme Sphingosine-1-phosphate Lyase1 (SGPL1) plays a key role in maintaining the right balance between S1P levels and other sphingolipid intermediates all of which affect cell growth, proliferation and cell death^23^. Indeed, uncontrolled up-regulation of SGPL1 results in a reduced availability of the bioactive lipid and a concomitant release of hexadecenal whose accumulation is cytotoxic *in vitro*^24^. Up-regulation of SGPL1 along with reduced levels of SPHK1 and subsequent decreased content of S1P has been found to be also associated with neurodegeneration in Alzheimer’s disease (AD)^25–27^.

Abnormal levels of both SPHK1 and 2 have been, indeed, described in experimental models of Parkinson’s disease (PD)^28^. Expression of SPHK1 has been reported to be significantly decremented also in a mouse model of HD^29^. Whether an imbalance in the S1P-metabolizing enzymes may further contribute to the worsening of the disease is still unclear, however, recent evidence demonstrates that either overexpression of SPHK1 or inhibition of SPHK2 is beneficial in an *in vitro* model of the disease^30,31^. Further support for the importance of this pathway to HD comes from the evidence that there is a reduced content of S1P in HD mouse striatal-derived cell lines (STHdh)^32^. Along this line, previous findings from our group and others have shown that administration of sphingolipids or sphingomimetic molecules mitigates the toxic effect of mHtt both in *in vitro* and *in vivo*^33–35^, all of which adds further weight to the hypothesis that aberrant sphingolipid metabolism is a key feature of the disease.

In summary, there is clear evidence for alteration of the sphingolipid metabolism in AD and PD^36–39^ but with only limited evidence to suggest this may also be the case in HD.

We therefore sought to further investigate whether deregulated metabolism of these lipids is a critical abnormality in HD using both animal models and patient samples. We report not only that such abnormalities are found in all cases and importantly early in the disease, but that pharmacological interventions aimed at modulating the activity of the sphingolipid metabolizing-enzymes may likely have disease-modifying effects *in vivo*.

## Material and Methods

### Human brain tissues

The “Cambridge Brain Bank” (Cambridge, UK) provided anonymous post-mortem brain samples (fresh frozen and paraffin-embedded tissues) from HD cases and healthy age-matched controls. All studies on human post-mortem brain tissues were approved by Neuromed Ethic Commitee (IRCCS Neuromed, RI 002012). The pathological severity of HD was scored according to the Vonsattel grading system^40^. Subjects’ clinical and genetic data are reported in Table 1. *Total lysate preparation* Tissues were homogenized in lysis buffer containing 20 mMTris, pH 7.4, 1% Nonidet P-40, 1 mM EDTA, 20 mMNaF, 2 mM Na_3_V0_4_, and protease inhibitor mixture (Santa Cruz, Cat N. sc-29131) and sonicated with 2 × 10 s pulses. *Immunohistochemistry*. Deparaffinized striatal and cortical tissue sections were soaked in 3% hydrogen peroxide to block endogenous peroxidase activity, incubated for 15 min in boiling citric acid buffer (10 mM, pH 6.0) and allowed to cool down at room temperature for 20 minutes. Slides were then washed in TBS and incubated with goat serum for 1 hour at room temperature. Sections were then incubated with anti-SGPL1 antibody (1:50) (Santa Cruz, Cat. N. sc-67368) overnight at 4 °C. Slides were washed in TBS and then incubated with biotinylated anti-rabbit IgG secondary antibody (Vectors Laboratories, Cat. N. BA-1000) for 1 hour at room temperature followed by horseradish peroxidase streptavidin (Vectors Laboratories, Cat. N. SA-5004). 3,3′-Diaminobenzidine tetrachloride (DAB) (Sigma Aldrich, Cat. N. D4293-50SET) was used for visualization. Control staining was performed without any primary antibody.

**Table 1:** Clinical and genetic data of human post-mortem brain tissues.

Cases		Age	Gender	Vonsattel’ neuropathology grade	CAGs (normal/expanded)
1	Healthy Control	67	M	CTRL	N/A
2	Healthy Control	75	M	CTRL	N/A
3	Healthy Control	72	M	CTRL	N/A
4	Manifest HD	66	M	III	17/45
5	Manifest HD	73	M	III	21/45
6	Manifest HD	68	M	IV	19/45

### Animal models

Mouse colonies were maintained in the animal facility at IRCCS Neuromed. All animal studies were performed in accordance with approved protocols by the IRCCS Neuromed Animal Care Review Board and by “Istituto Superiore di Sanità” (permit number: 1163/2015- PR) and were conducted according to EU Directive 2010/63/EU for animal experiments. All the analyses were performed on manifest early manifest (6 week old) and on manifest (11 week old) R6/2 and manifest (9 month old) YAC128 HD mice and age-matched wild-type (WT) littermates. R6/2 mouse model, overexpressing the exon 1 of the human HD gene (*HTT*) with more than 150 CAG-repeat-expansion^41,42^, is one of the best-characterized and the most widely used animal model which recapitulates many of the features of HD human pathology and frequently used in preclinical studies. Motor symptoms usually start at 6 weeks of age and progressively worsen over the weeks^41,42^. YAC128 mice represent another best-characterized animal model of HD, expressing the entire human HD gene (including promoter region) with 128 CAG repeats. Even YAC128 mice display an array of motor and neuropathological changes that largely recapitulate the human pathology^43^. Hyperkinesia begins at 3 months of age with progressive motor impairment appearing at 6 months of age.

#### Total lysate preparation

Mice were sacrificed by cervical dislocation and the entire brain regions (striatum and cortex) were dissected out, snap frozen in liquid N2 and pulverized in a mortar with a pestle and the lysate obtained as reported above.

### Mass Spectrometry

Snap frozen brain tissues were transferred into glass tubes and stored at −80 °C until lipid extraction. Lipids were extracted using a modified Bligh and Dyer method. Briefly, brain tissues were homogenized in 2 mL CHCl_3_/MeOH (1:2, v/v) with 0.1% TFA containing internal standards. Then 600 µL of both chloroform and water were added with intermittent mixing for 30 sec. The samples were then centrifuged for 10 min at 3000 rpm at 4 °C. The organic extracts were divided into two equal parts. One part was evaporated under nitrogen stream and reconstituted in 100 µL MeOH/CHCl_3_ (9:1, v/v) for LCMS analysis of Sphingomyelins, Sphingolipid bases and their phosphate metabolites. The other part was fractionated by Silica Gel G (60-Å 230–400 Mesh ASTM; Sigma-Aldrich, Milan, Italy) column chromatography. Ceramides were eluted with 2 ml of CHCl_3_/MeOH (9:1, v/v), then hexosylceramides were eluted with 1 ml of CHCl_3_/MeOH (8:2, v/v). Both the fractions were pooled together and evaporated under nitrogen stream and dried pellets were reconstituted in 100 µl of MeOH/CHCl_3_ (9:1, v/v) and transferred to glass vials for LC/MS analyses. LC-MS/MS analyses of the samples were carried out on the Acquity UPLC system coupled with a Xevo TQ-MS triple-quadrupole mass spectrometer as previously described^44^.

### Analysis of ceramide by immunohistochemistry

For analysis of ceramide, mice were decapitated and the entire brain removed and frozen in cool isopentane (−80 °C). Each brain was cut serially with a Jung CM1900 Cryostat (Leica Instruments, Germany) in 20 μm thick sections. Brain sections were incubated with anti-Ceramide (MID 15B4) (1:200) (Enzo, Cat. N. ALX-804-196; lot number: 06061610)^45^ over-night at 4 °C and then with biotinylated anti-mouse IgG secondary antibody (Vectors Laboratories, Cat. N. BA-2000) for 1 hour at room temperature followed by horseradish peroxidase streptavidin (Vectors Laboratories, Cat. N. SA-5004). 3,3′-Diaminobenzidine tetrachloride (DAB) (Sigma Aldrich, Cat. N. D4293-50SET) was used for detection.

### Analysis of ceramide by Dot Blotting

After tissue lysis protein quantitation was first assessed by Bradford method. Next, to assure that equal amount of homogenate was analyzed, each sample tissue lysate was serially diluted and protein concentration was re-assessed by NanoDrop Spectrophotometer^9^. Fifty nanograms of total protein lysates, from HD and control mice were then spotted in quadruplicates on nitrocellulose membrane and, dot-blotting analysis was performed as previously reported^9^. Ceramides was detected with anti-ceramide (1:200) (Enzo, Cat. N. ALX-804-196; lot number: 06061610). A goat anti-mouse HRP-conjugated secondary antibody (1:5000) (Santa Cruz, Cat. N. sc-2005; lot number: B0813) was used. Ceramide spots were detected by ECL Prime (GE Healthcare) and quantitated with Quantity One (Bio-Rad Laboratories).

### Chemicals

(2R, 3S, 4E)-N-methyl-5-(4′-pentylphenyl)-2-aminopent-4-ene-1,3-diol (SK1-I) (Enzo, Cat. N. BML-EI411); 2-acetyl-4-(tetrahydroxybutyl)-imidazole (THI**)** (Cayman, Cat. N. 13222), K145 (Sigma-Aldrich, Cat. N. SML1003-5MG), EMD567731 (Cayman, Cat. N. 567731), N,N-Dimethylsphingosine (DMS) (Enzo, Cat. N. BML-SL105) and K6PC-5 (NeoPharm, Korea) were dissolved according to the manufacturer’s instruction.

### Cell models

Conditionally immortalized mouse striatal knock-in cells expressing endogenous levels of wild-type (STHdh^7/7^) or mHtt (STHdh^111/111^) were purchased from the Coriell Cell Repositories (Coriell Institute for Medical Research, Camden, NJ, USA) and maintained as previously described^4^. *Lysate preparation*. Cells were lysed in lysis buffer as reported above.

### Human iPSC-derived neurons

Neurons were differentiated from previously published representative HD hiPSC lines, HD58-3 (58 CAG-repeat expansion), that have been validated to be pluripotent and exhibit cellular features consistent with HD^46^. Cortical glutamatergic differentiation was done using a 11day dual SMAD neural induction protocol, as previously described^47,48^, except that LDN (4 µM) (Stemgent Cat. N. 04-0074) and SB431542 (10 µM) (Stemgent Cat. N.04-0010) was added to the medium throughout the 11 days. Neural induction was followed by terminal differentiation and maturation in neural differentiation medium consisting of a 1:1 mixture of N-2 medium (DMEM/F-12 GlutaMAX, N-2 supplement (1x), 100 µm nonessential amino acids, 100 μM 2-mercaptoethanol, 2000 U/ml penicillin and 2000 µg/ml streptomycin) and B-27-containing neurobasal medium (Neurobasal medium, B-27 supplement (1x), 2 mM Glutamax)^49,50^. We have previously confirmed expression (protein and/or mRNA) of cortical glutamatergic markers (e.g. vGlut1, glutamate, Sox1, Pax6, FoxG1, synapsin, homer along appropriate) using this protocol (data not shown). By day 33 in culture, neuronal processes positive for ß3-Tubulin and MAP2 were observed (Supplementary Fig. 1). HD58-3 hiPSC-derived cortical neurons were further differentiated to day 63. On day 63 of differentiation, the neurons were treated with accutase (Innovative Cell Technologies Cat. N. AT-104) and re-plated at a density of 1 million cells per 10 cm^2^ in neural differentiation medium containing 10 µM Rock-inhibitor. In K6PC-5 experiments, HD58-3 hiPSCs were re-plated from independent wells to provide three experimental replicates. On day 64, the media was changed to remove the ROCK inhibitor and the cortical neurons exposed on day 65 to 50 µM K6PC-5 for 30 minutes. *Lysate preparation*. Neuronal cultures were washed once with ice cold PBS and then lysed in 100 µl of RIPA buffer containing protease (Sigma- Aldrich Cat. N. P8340) and phosphatase inhibitor cocktails 2 and 3 (Sigma-Aldrich Cat. N. P5726, Cat. N. P0044), and the lysates centrifuged at 4 °C for 20 min at 20,000 g. The protein concentration of the resulting supernatant was quantified using the Pierce BCA Protein Assay (Thermo Scientific). Twenty-five micrograms of neuronal proteins were loaded for each sample on a 4–20% pre-cast SDS-PAGE gel (BioRad) and run for 3 hours at 90 V. Proteins were then transferred onto nitrocellulose membranes using iBlot Gel Transfer Device (Life Technologies) and the gel was stained with Coomassie (BioRad) for 1 hour. The nitrocellulose membrane was blocked in Odyssey Blocking Buffer for 1 hour, and then incubated with anti phospho-AKT and AKT and anti phospho-ERK and ERK antibodies as reported below. Protein bands were detected by ECL Prime (GE Healthcare) and quantitated with Quantity One Software (Bio-Rad Laboratories).

### Immunoblottings

For SGPL1, SPHK1, SPHK2, twenty-five micrograms of total protein lysate were immunoblotted with the following antibodies: anti-SGPL1 (1:1000) (Santa Cruz, Cat. N. sc-67368), anti-SPHK1 (1:1000) (Abcam, Cat. N. ab71700; lot numbers: GR17790-4 and GR17790-24) and anti-SPHK2 (1:1000) (Abcam, Cat. N. ab37977; lot numbers: GR31063-12 and GR31063-39). For phospho-AKT, AKT, phospho-ERK and ERK, total lysate was immunoblotted with the following antibodies: anti-phospho-AKT (1:1000) (Cell Signaling, Cat. N. 9271), anti-AKT (1:1000) (Cell Signaling Cat. N. 2920), anti-phospho-ERK (1:1000) (Cell Signaling Cat. N. 9101), anti-ERK (1:1000) (Cell Signaling Cat. N. 9102), and anti-ERK (1:1000) (Cell Signaling Cat. N. 4696). For protein normalization, anti-Actin (1:5000) (Sigma Aldrich, Cat. N. A5441) or anti-Cyclophilin (1:3000) (Abcam, Cat. N. ab16045) was used. Protein bands were detected by ECL Prime (GE Healthcare) and quantitated with Quantity One Software (Bio-Rad Laboratories).

### RNA extraction and analysis of gene expression by real-time PCR

Total RNA was extracted using RNeasy kit (Qiagen) according to the manufacturer’s instructions. One microgram of total RNA was reverse-transcribed using Superscript II reverse transcriptase (Invitrogen) and oligo-dT primer, and the resulting cDNAs were amplified using Power SYBR Green PCR Master Mix (Applied Biosystems) following the manufacturers’ instructions. Quantitative PCR analysis will be performed on a StepOne instrument (Applied Biosystems) using specific the following primers:

mouse SGPL1 Fw: 5′-cattcgacaaagcagctcat-3′;

mouse SGPL1 Rev: 5′-ctcttcattgcctgcacatc-3′;

mouse SPHK1 Fw: 5′-tgtgaaccactatgctgggta-3′;

mouse SPHK1 Rev: 5′-cagcccagaagcagtgtg-3′;

mouse SPHK2 Fw: 5′-agacgggctgctttacga-3′;

mouse SPHK2 Rev: 5′-caggggaggacaccaatg-3′;

mouse Cyclophilin Fw: 5′-tccaaagacagcagaaaactttcg-3′;

mouse Cyclophilin Rev: 5′-tcttcttgctggtcttgccattcc-3′.

### Analysis of apoptosis

Apoptosis was assessed as previously described^34^. In THI experiments, cells were cultured in standard growth medium for three days in the presence and absence of THI, then they were placed at 39 °C for five hours in SFM. In K145, EMD567731, SK1-I and DMS experiments, cells were pre-treated with each inhibitor at 33 °C for 1 hour in serum-free medium (SFM) and then placed at 39 °C for five hours. In K6PC-5 experiments, cells were incubated in SFM and placed at 39 °C for five hours in the presence and absence of the SPHK1 activator. At the end of each treatment, cells were collected and incubated with FITC-conjugated Annexin V (BD, Cat. N. 556419), according to the manufacturer’s instructions. Fluorescence-activated cell sorting (FACS) analysis was performed as previously described^34^.

### Statistics

Two side Paired and Unpaired t-tests were used as indicated. Data are expressed as mean ± SD.

## Results

### Expression of sphingolipid-metabolizing enzymes is aberrant in HD human post-mortem brains

**Figure 2:**
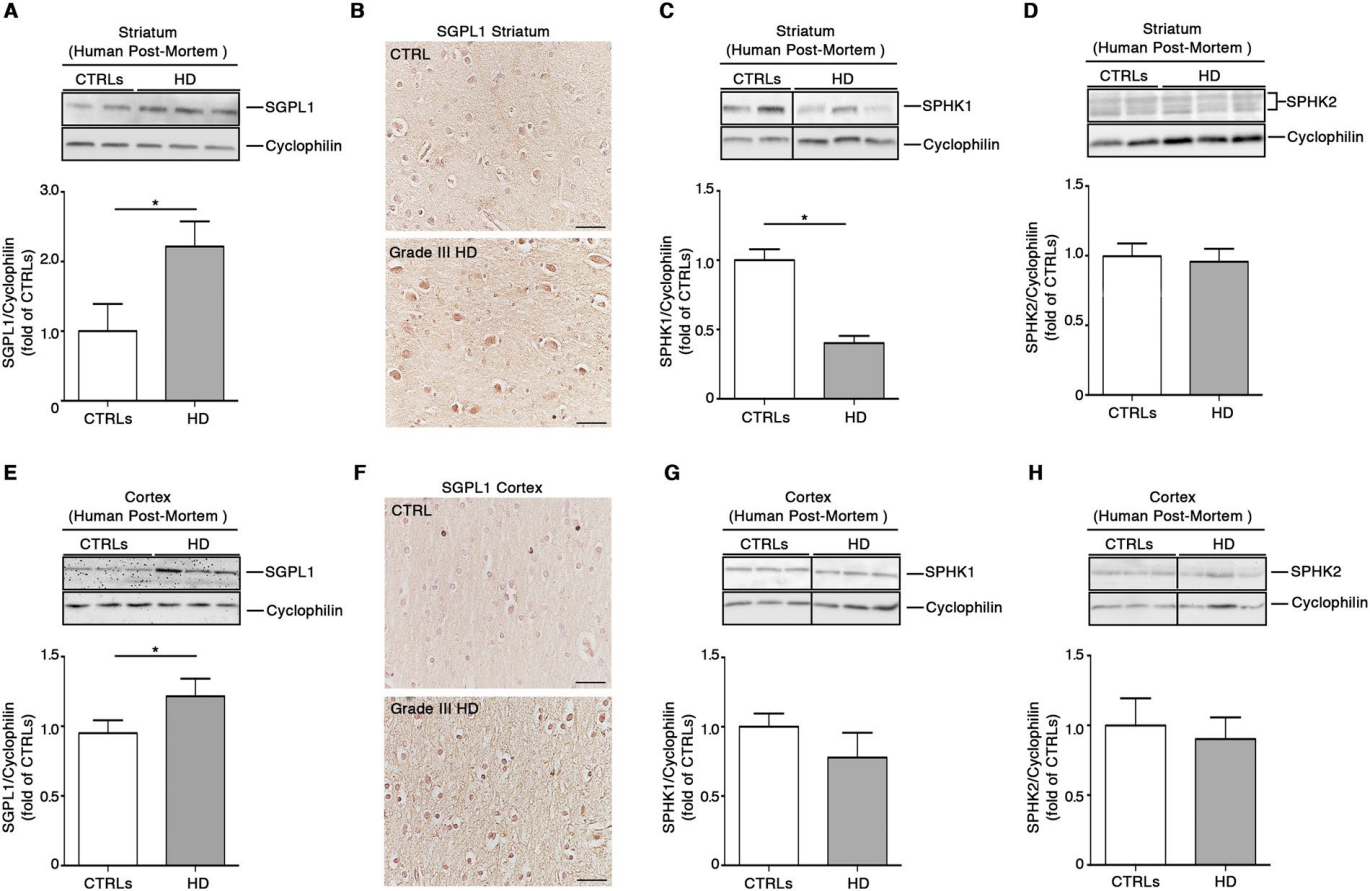
Expression of S1P metabolizing enzymes is abnormal in post-mortem brain tissues from HD patients. Cropped immunoblottings and densitometric analysis (**A**) along with representative immunohistochemical micrographs (**B**) of SGPL1 expression in the striatum from post-mortem human brains of manifest HD patients and age- and gender-matched healthy controls (see Table 1). Cropped immunoblottings and densitometric analysis of SPHK1(**C**) and SPHK2 isoform expression (**D**) in the striatum from post-mortem human brains of the same subjects. Cropped immunoblottings and densitometric analysis (**E**) along with representative immunohistochemical micrographs (**F**) of SGPL1 in the cortex from human post-mortem brains of manifest HD patients and age- and gender-matched healthy controls. Cropped immunoblottings and densitometric analysis of SPHK1(**G**) and SPHK2 isoform expression (**H**) in the cortex from post-mortem human brains of the same subjects. Scale bar in each micrograph represents 100 µm. In each immunoblotting, all samples were run on the same gel. Non-adjacent samples were separated by a black line. Values are mean ± SD. N = 2–3 for each group of patients. *p < 0.05 (Unpaired t-test).

Deregulated expression and activity of sphingolipid-metabolizing enzymes have been recently described in animal models and patients with a range of chronic neurodegenerative conditions^25,26,28,51^. Here, in order to investigate whether similar biochemical defects also occur in HD, we analyzed post-mortem striatal and cortical specimens from patients with advanced HD (grade III-IV)^40^ compared to gender and age-matched healthy controls (Table 1). Immunoblotting analysis showed that the expression of SGPL1 was robustly increased in both the striatum and cortex of these HD patients (Fig. 2A and E) and this was further confirmed by immunohistochemistry (Fig. 2B and F). In line with the hypothesis that there is a major disturbance in the regulation of either the synthesis or degradation of sphingolipids, increased SGPL1 was associated with a significant reduction of SPHK1 in the striatum of the same HD patients when compared to healthy controls (Fig. 2C). Although a trend towards a similar reduction of SPHK1 protein expression in the cortex was seen, this did not reach statistical significance (Fig. 2G). No changes in the expression profile of multiple isoforms^52–54^ of SPHK2 could be detected neither in the striatum nor in the cortex (Fig. 2D and H).

### S1P-metabolizing enzymes in HD animal models showed an expression profile similar to that seen in the HD human post-mortem samples

In order to clarify whether alterations in the sphingolipid metabolic pathways are relevant to the neurodegenerative process in HD, we examined brain tissues from two different HD mouse models (YAC128 and R6/2 mice).

**Figure 3:**
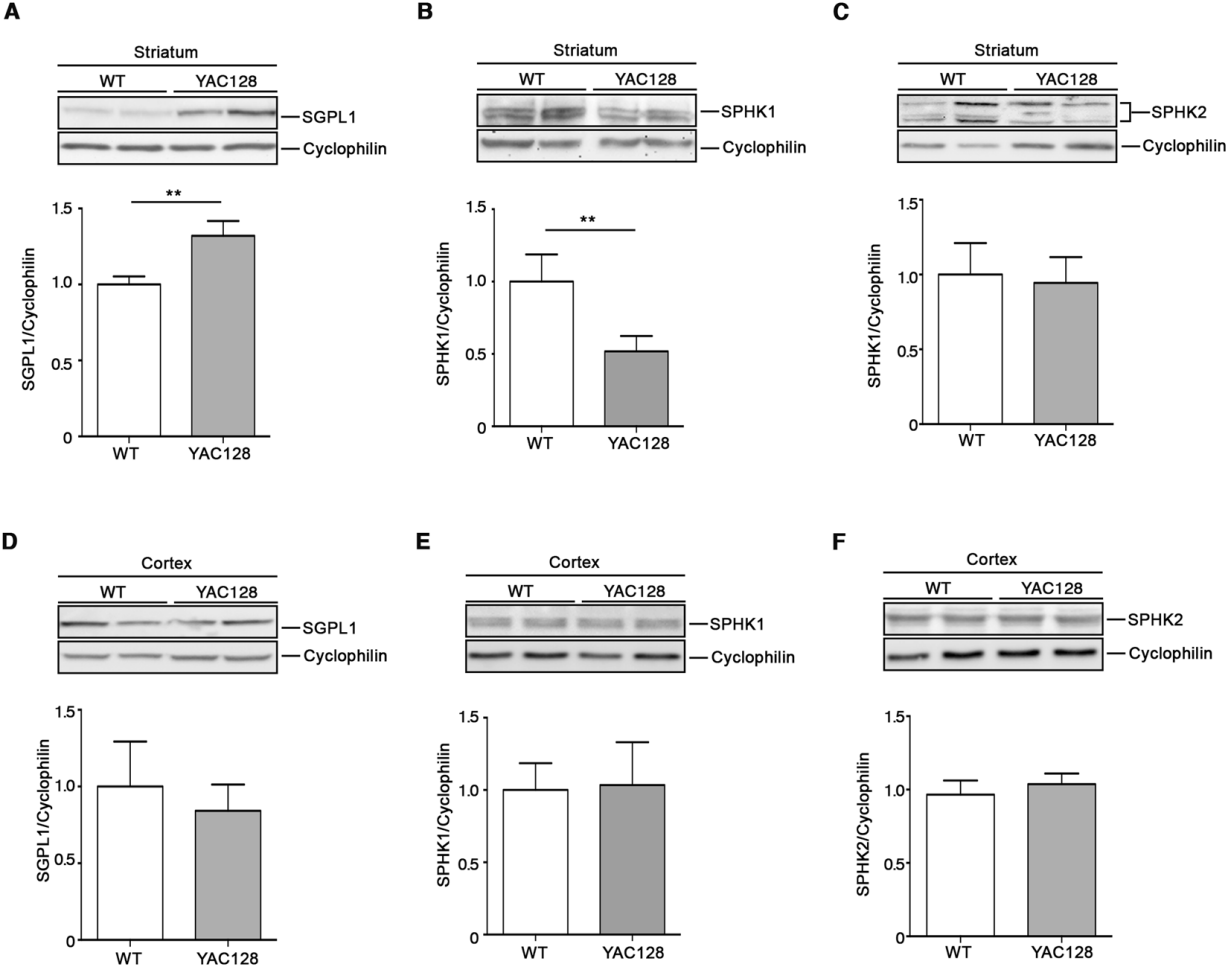
Expression of S1P-metabolizing enzymes is defective in the striatum of YAC128 HD mice. Representative cropped immunoblottings and densitometric analysis of SGPL1 and SPHK1/2 isoform expression in striatal (**A–C**) and cortical (**D,E**) tissues from manifest (9 month old) YAC128 mice and WT littermates. Value are represented as mean ± SD. N = 5 for each group of mice. *p < 0.05; **p < 0.001 (Unpaired t-test).

Biochemical analysis of all three SGPL1, SPHK1 and SPHK2 proteins, in the striatal tissues from manifest YAC128 mice, showed expression profiles similar to that in human tissues (Fig. 3A–C). While expression of SGPL1 was significantly increased (Fig. 3A), levels of multiple isoforms^52,53,55^ of SPHK1 were markedly reduced in these HD mice when compared to wild-type (WT) littermates (Fig. 3B). No changes in the expression of SPHK2 isoforms were, indeed, observed (Fig. 3C). When cortical tissues were analyzed, no variation was observed in any of the three proteins (Fig. 3D–F).

**Figure 4:**
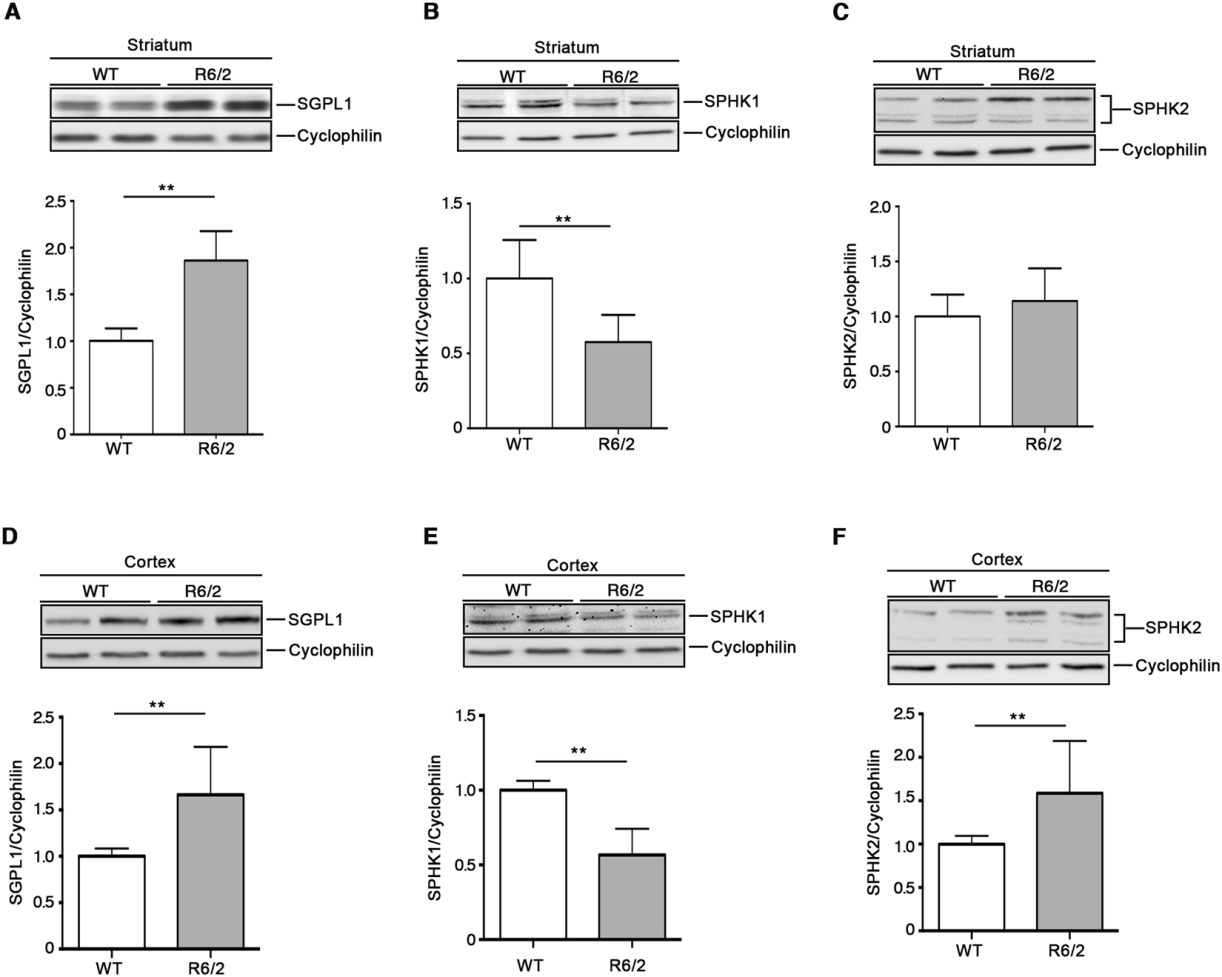
Expression of S1P metabolizing enzymes is defective in brain tissues from R6/2 HD mice. Representative cropped immunoblottings and densitometric analysis of SGPL1 and SPHK1/2 isoform expression in striatal (**A–C**) and cortical (**D,E**) tissues from manifest (11 week old) R6/2 mice and WT littermates. Values are represented as mean ± SD. N = 6–9 for each group of mice. **p < 0.001 (Unpaired t-test).

To further investigate the aberrant sphingolipid metabolism in HD, biochemical analyses were extended to brain tissues from manifest R6/2 mice (Fig. 4) which express exon 1 of human HTT with over 150 poly-Q. Importantly, protein expression profiles of either SGPL1 or SPHK1 in these mice mirrored the pattern seen in YAC128 mice and human samples in both striatum and cortex (Fig. 4A,B). Indeed, SPHK2 expression showed a slight, but not significant trend toward the increase in the striatum (Fig. 4C) and was considerably incremented in the cortex (Fig. 4F).

### Brain tissues of manifest R6/2 mice showed perturbed bioavailability of essential sphingolipids

**Figure 5:**
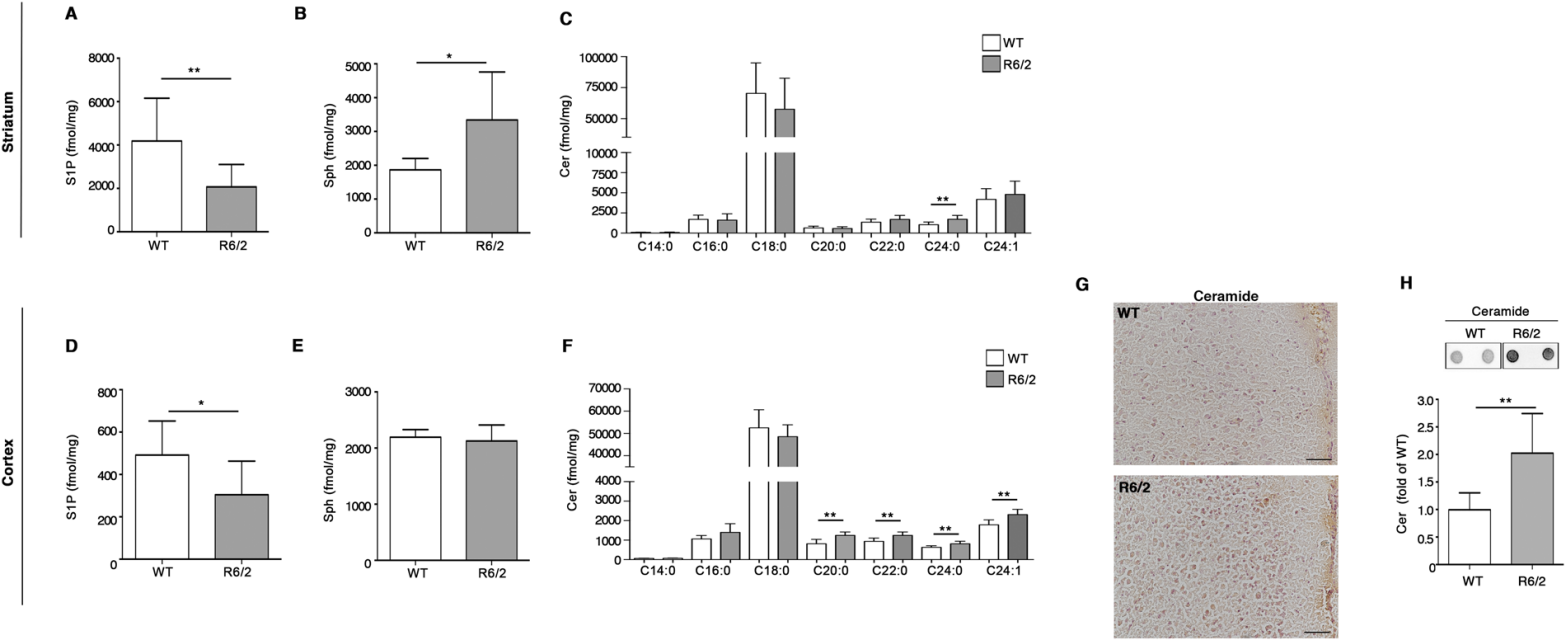
Levels of essential sphingolipids are defective in brain tissues from R6/2 mice. Lipidomic analysis by LC-MS/MS of S1P, Sph and Cer content in striatal **(A–C)** and cortical **(D,F)** tissues from manifest (11 week old) R6/2 mice and WT littermates. Values are represented as mean ± SD. N = 7–10 for each group of mice. *p < 0.05; **p < 0.001; ***p < 0.0001 (Unpaired t-test). Representative immunohistochemical micrograph (**G**) and dot blotting and densitometric analysis (**H**) of Cer content in the cortex of manifest R6/2 mice and WT littermates. Scale bar in each micrograph represents 100 µm. Data are represented as mean ± SD. N = 5 for each group of mice. **p < 0.001 (Unpaired t-test).

Expression levels of either SGPL1 or SPHKs have been previously associated with an altered bioavailability of the bioactive sphingolipid, S1P^56,57^. In order to verify whether a similar association exists in HD, sphingolipid content in R6/2 mouse brains was determined by a targeted sphingolipidomic analysis^44^. Consistent with the immunoblotting analyses, mass spectrometry revealed a significant reduction of S1P content in both striatum and cortex of manifest R6/2 mice (Fig. 5A and D).

**Figure 6:**
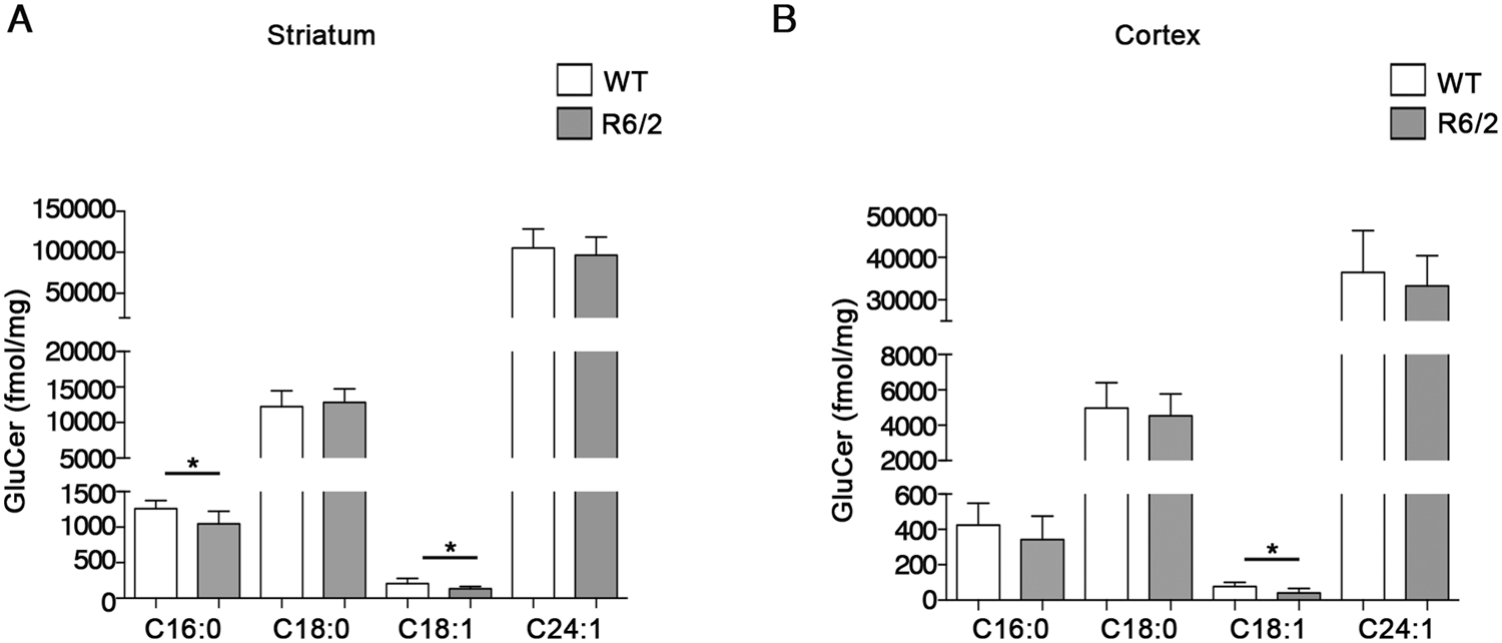
Levels of Glucosylceramides (GluCer) are reduced in brain tissues from R6/2 mice. Lipidomic analysis by LC-MS/MS of GluCer content in the striatal (**A**) and cortical (**B**) tissues from manifest (11 week old) R6/2 mice and WT littermates. Values are represented as mean ± SD. N = 7 for each group of mice. **p < 0.001(Unpaired t-test).

Beside the defective expression of the metabolizing enzymes, SPGL1 and SPHKs (Fig. 4), reduced levels of S1P in the brain tissues of R6/2 mice may be attributable to altered levels of Sph or to an overall imbalanced sphingolipids rheostat. Accordingly, quantitative analysis by Mass Spectrometry highlighted a significant increase of Sph in the striatum of R6/2 mice (Fig. 5B), however no changes were observed in the cortex (Fig. 5E). Mass spectral data revealed also a complex Cer profile in these two specific brain regions. Although the striatal tissues displayed increased levels of only one Cer species (C24:0), cortical tissues showed accumulation of different molecular species of the same lipid (C20:0, C22:0, C24:0 and C24:1) (Fig. 5C–F). Accumulation of Cer in the cortex was confirmed by immunohistochemical and dot blotting analysis (Fig. 5G–H). This accumulation was also associated with a reduction of some GluCer species as shown in Fig. 6.

### Sphingolipid metabolism is aberrant at early stage of the disease in brain tissues from R6/2 mice

**Figure 7:**
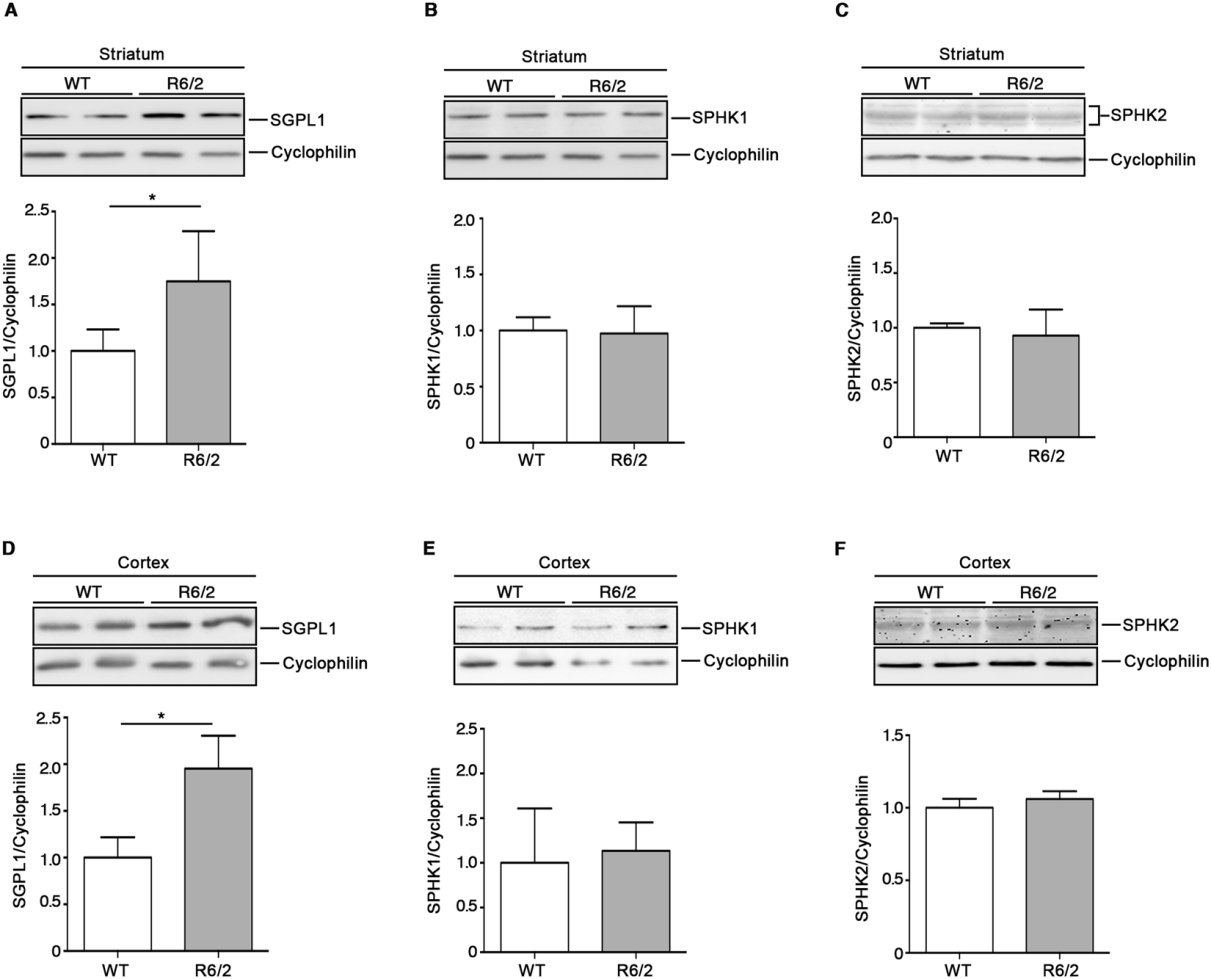
Levels of both SGPL1 are defective in brain tissues from R6/2 mice at early stages of the disease. Representative cropped immunoblottings and densitometric analysis of SGPL1 and SPHK1/2 isoform expression in striatal (**A**–**C**) and cortical (**D**,**E**) tissues from early manifest (6 week old) R6/2 mice and WT littermates. Values are represented as mean ± SD. N = 5 for each group of mice. **p < 0.001 (Unpaired t-test).

**Figure 8:**
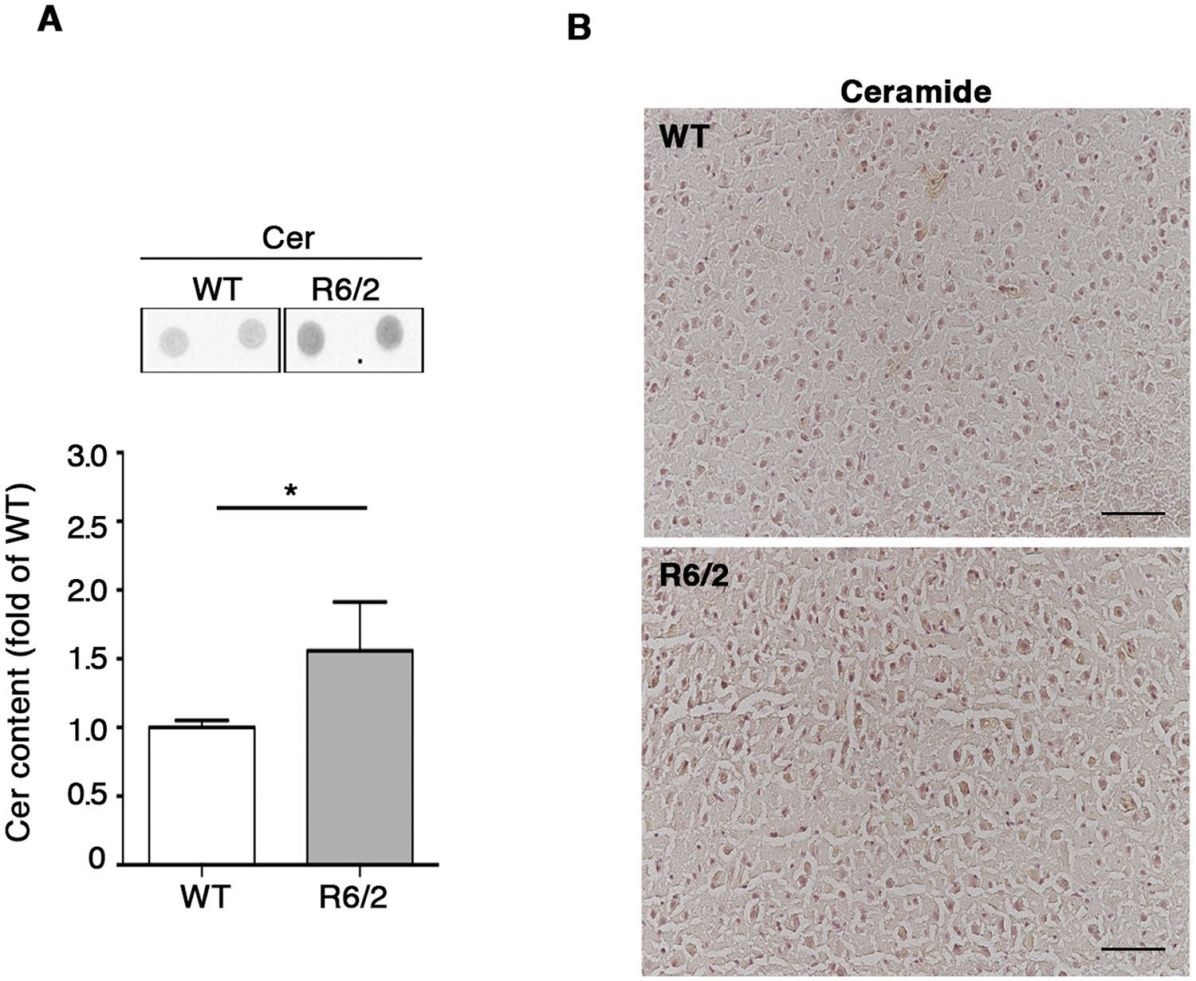
Levels of ceramides are increased in cortical tissue from R6/2 mice at early stages of the disease. Representative dot blotting and densitometric analysis (**A**) and immunohistochemical micrograph. Scale bar in each micrograph represents 100 µm. (**B**) of Cer content in the cortex of early manifest (6 week old) R6/2 mice and WT littermates. Data are represented as mean ± SD. N = 5 for each group of mice. *p < 0.05 (Unpaired t-test).

In order to assess whether alteration of sphingolipid metabolism may represent an early biological event, potentially underlying HD pathophysiology, we investigated the expression of S1P-metabolizing enzymes in both striatal and cortical tissues from early manifest (6 week old) R6/2 mice. Importantly, immunoblotting analysis showed a significant increase of SGPL1 levels in both regions already at such early stage of the disease. No difference in SPHK1 and 2 expression was indeed detectable (Fig. 7A–F). Interestingly, semi-quantitative analysis by dot blotting pointed out also a robust increase of Cer content in the cortex of the same mice (Fig. 8A). This result was confirmed by immuhistochemical analysis (Fig. 8B).

### Pharmacological modulation of S1P-metabolizing enzyme activity regulates pro-survival pathways in HD cells

Modulation of sphingolipid metabolizing-enzyme activity has been widely described to alter the bioavailability of S1P and by so doing cellular homeostasis and survival^58,59^.

**Figure 9:**
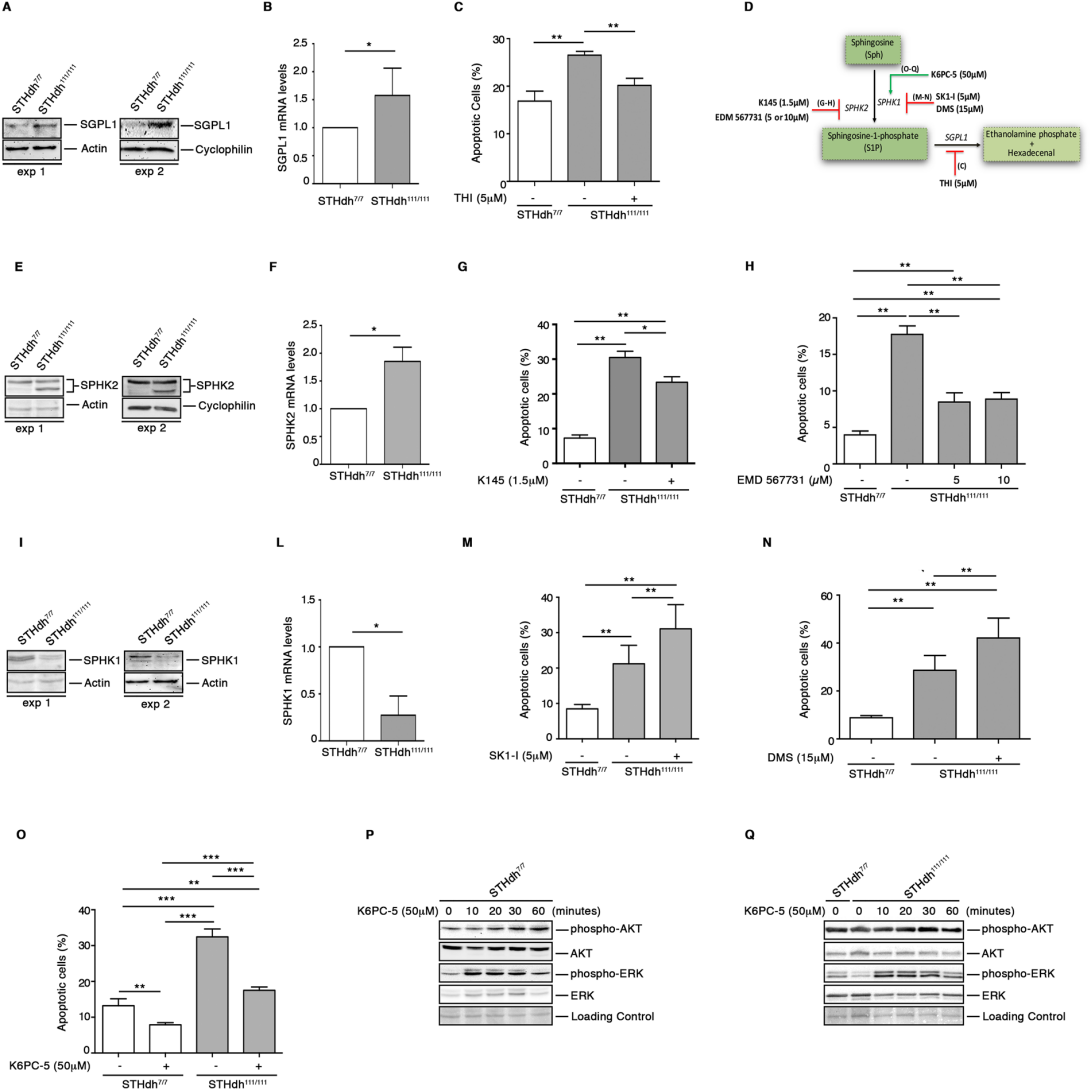
Pharmacological modulation of S1P metabolizing enzyme activity regulates cell survival in HD striatal derived cells. Cropped immunoblotting of SGPL1 protein (**A**) and mRNA expression levels (**B**) from STHdh^7/7^ and STHdh^111/111^ cells. Values are mean ± SD of three experiments performed in triplicate. *p < 0.05 (Unpaired t-test). Analysis of apoptosis in STHdh cell lines first cultured for three days in complete medium in the absence and presence of 5 μM THI and then incubated for five hours in serum free medium to induce apoptosis (**C**). Values are mean ± SD of two experiments performed in quadruplicate. **p < 0.001(Unpaired t-test). Schematic representation of the pharmacological interventions to modulate the activity of S1P-metabolizing enzymes (**D**). Cropped immunoblotting of SPHK2 protein (**E**) and mRNA expression levels (**F**) from STHdh^7/7^ and STHdh^111/111^ cells. Values are mean ± SD of three experiments performed in triplicate. *p < 0.05 (Unpaired t-test). Analysis of apoptosis in STHdh cell lines incubated for five hours in serum free media in the presence or absence of either 1.5 μM K145 (**G**) or 5 and 10 μM EMD567731 (**H**) SPHK2 inhibitors. Values are mean ± SD of three experiments, each performed in triplicate. *p < 0.05; **p < 0.001 (Unpaired t-test). Cropped immunoblotting of SPHK1 protein (**I**) and mRNA expression levels (**L**) from STHdh^7/7^ and STHdh^111/111^ cells. Values are mean ± SD of three experiments performed in triplicate. *p < 0.05 (Unpaired t-test). Analysis of apoptosis in STHdh cell lines incubated for five hours in serum free media in the presence or absence of 5 μM SK1-I (2R, 3S, 4E)-N-methyl-5-(4′-pentylphenyl)-2-aminopent-4-ene-1,3-diol) (**M**) and 15 μM DMS (N,N-Dimethylsphingosine) (**N**). Data are mean ± SD of three experiments, each performed in triplicate. **p < 0.001 (Unpaired t-test). Apoptosis in STHdh cell lines incubated for five hours in serum-free medium in the presence or absence of 50 μM K6PC-5 (**O**). Data are mean ± SD of three experiments, each performed in triplicate. **p < 0.001; ****p < 0.0001 (Unpaired t-test). Cropped immunoblotting of AKT and ERK phosphorylation in cellular protein extracts from STHdh^7/7^ (**P**) and STHdh^111/111^ (**Q**) cell lines measured at different time points after K6PC-5 treatment.

In order to investigate the potential role of all three S1P-metabolizing enzymes in HD cellular homeostasis, we undertook pharmacological studies (Fig. 9D) in mice striatal-derived knock in cell lines expressing endogenous levels of wild type (STHdh^7/7^) or mHtt (STHdh^111/111^) and where levels of S1P have been previously reported to be reduced^32^.

In line with our earlier data, both protein and gene expression of these enzymes was markedly abnormal in these cells (Fig. 9A,B,E,F and I–L). While higher levels of SGPL1 in the mHtt cells were associated with an increased susceptibility to apoptosis (Fig. 9A–C), its pharmacological inhibition by 2-acetyl-4-(tetrahydroxybutyl) imidazole (5 μM THI) re-established the vulnerability of these cells to cell death to the extent that they behaved as WT cells (Fig. 9C). Increased expression of SGPL1 was paralleled by visibly higher levels of SPHK2 (Fig. 9E,F), and according to other studies^31^, its inhibition by either K145 (1.5 μM) or EMD567731 (5 and 10 μM) significantly protected cells form apoptosis (Fig. 9G,H).

Contrary to what was found for SGPL1 and SPHK2, levels of SPHK1 were significantly reduced in HD cells (Fig. 9I–L). Although the impact that such an imbalance may have on cell function is not clear yet, pharmacological inhibition of SPHK1 by either SK1-I (5 μM) or DMS (15 μM) further exacerbated the susceptibility of HD cells to apoptosis (Fig. 9M,N).

**Figure 10:**
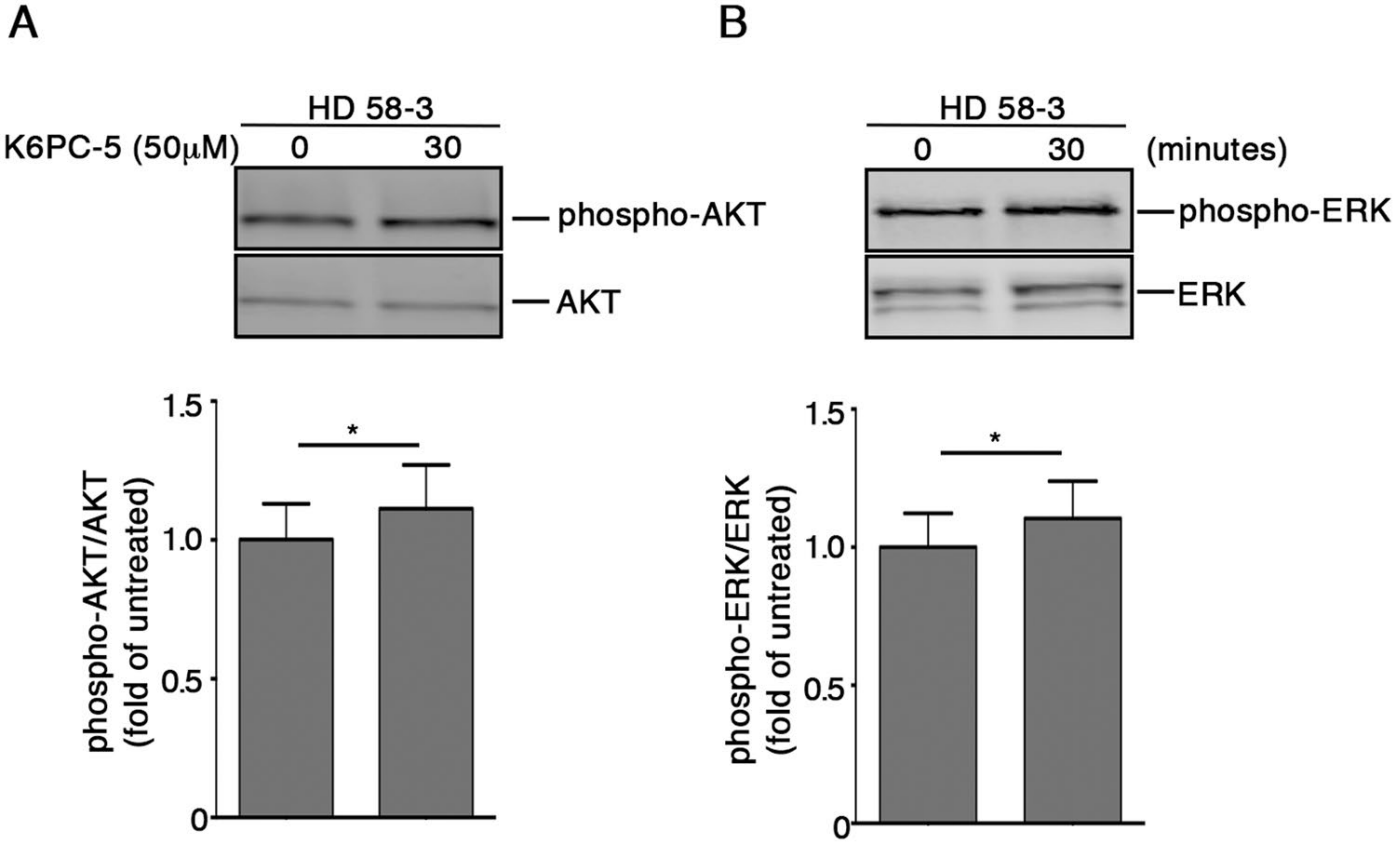
Pharmacological activation of SPHK1 evokes activation of pro-survival pathways in human iPSC-derived neurons. Representative cropped immunoblottings and densitometric analysis of AKT (**A**) and ERK (**B**) phosphorylation in iPSC-derived HD human neurons (day 65) after 30 minute of treatment with K6PC-5. Data are mean ± SD of three experiments, * p < 0.05 (Paired t-test).

Finally, with the aim of elucidating the extent to which SPHK1 may influence cell survival in the presence of mHtt, we evaluated the potential beneficial effect of its pharmacological activation by using K6PC-5, a selective stimulator of SPHK1 activity^60–62^. Importantly, treatment with 50 μM K6PC-5 significantly mitigated the vulnerability of cells to apoptosis (Fig. 9O) and rapidly induced the activation of either AKT or ERK in both WT and HD cells as shown in Fig. 9P,Q. Importantly, administration of K6PC-5 triggered a slight, but significant increase of both phospho-AKT and phospho-ERK also in iPSC-derived cortical neurons from HD patients (Fig. 10A,B and Supplementary Fig. 2).

## Discussion

There is increasing evidence that links perturbed sphingolipid metabolism to a range of different neurodegenerative conditions including HD^4,25,26,28^.

In this study, we consolidated the evidence that sphingolipid metabolism is aberrant in HD and represents a common pathogenic denominator among multiple preclinical models of the disease and in human patients. Also, we demonstrated for the first time, that expression of S1P-metabolizing enzymes is significantly altered. In particular, we have found that such a defect shows some regional variation in the HD brain, with especially consistent expression profile abnormalities seen between SPGL1 and SPHK1 in the striatal tissues of all our HD human samples and animal models. Up-regulation of SGPL1 was consistently paralleled by down-regulation of SPHK1, which, under physiological conditions, is known to act as a pro-survival kinase in both CNS and peripheral tissues^18,63,64^.

The role of SPHK2 in HD, indeed, remains elusive. The significance of the differential expression of SPHK2 between brain tissues from R6/2 mice and YAC128 mice and HD patients is not straightforward, however it may be that SPHK2 has different roles depending on when and where it is expressed or on its subcellular localization^65^. Very recent findings reported a detrimental effect of SPHK2 in different *in vitro* models of the disease^65^. Thus, we speculate that elevation of its expression in brain tissues from R6/2 mice may be toxic and further contribute to the worsening of the disease in this mouse model.

Alteration in the expression of S1P-metabolizing enzymes may theoretically correlate with a defective availability of the bioactive lipids in HD. Accordingly, lipidomic analysis revealed a significant decrease in the levels of S1P in both striatum and cortex of manifest R6/2 mice. Our study also provides the first *in vivo* evidence of perturbed ceramide homeostasis, whose increased levels have been described to interfere with mitochondrial channel formation, exert pro-apoptotic actions^66,67^ and to be associated with different neurodegenerative conditions^39,68,69^.

Where the reduction of S1P content in the brain tissues of R6/2 mice comes from, is currently unclear. However, taking into account the abnormal increase of Sph levels in the striatum along with accumulation of different Cer species in the cortex of these mice and, considering also the increased levels of S1P-degradative enzyme, SGPL1, in both brain regions, disturbed S1P homeostasis may be due to a combination of poor conversion of Cer to S1P, through Sph production and/or to its enhanced degradation.

Importantly, abnormal expression of SGPL1 and higher levels of Cer, already detectable at early stage of disease in R6/2 mice, likely suggest that S1P metabolism is precociously affected in HD.

This finding corroborated our previous evidence demonstrating that perturbations of the sphingolipid metabolism occur early in the disease^9^. Moreover, the absence of defects in the expression of both SPHK1 and 2 proteins in early manifest mice also indicates that such alterations may progress with the worsening of the disease.

Although not investigated how huntington mutation may alter the expression of S1P-metabolizing enzymes,﻿ its early occurrence may likely be attributable to gene expression deregulations, as they may take place before disease onset^70^. To this regard, evidence indicates that SGPL1 gene expression is usually negatively regulated by the micro RNA - miRNA 125b^71^. Interestingly, expression of miRNA 125b is described being down-regulated in different pre-clinical models of HD including R6/2 mice^72^. In the light of that, we hypothesize that SGPL1 over-expression may be likely attributable to micro RNA dysregulation. However, our findings suggested that aberrant enzyme protein content likely depends on altered gene expression.

From our perspective, our findings provide first insights into perturbed S1P metabolism, which does not represent an epiphenomenon connected to the worsening of the disease, but rather a mutation-dependent biological event with a potential pathogenic role in HD.

Along this line, pharmacological interventions aimed at modulating S1P metabolism may pave the way for the development of more targeted and effective therapeutic strategies for the treatment of HD. Our results are in line with recent evidence showing the potential beneficial effect of modulation of SPHKs *in vitro*^30,31^, and strengthen our previous findings demonstrating a neuroprotective effect of the S1P receptor stimulation *in vivo*^34^. Importantly, the beneficial effects of pharmacological modulation of S1P-metabolizing enzymes in HD human iPSC-derived neurons, further support our hypothesis.

We believe that the novelty of our study lies in the evidence that alteration in S1P metabolism may represent a new hallmark of the disease as it is shared amongst preclinical models of HD and most importantly visible also in human post-mortem brains. In addition, its early occurrence corroborates the hypothesis that defective sphingolipid homeostasis may likely contribute to the development of HD. In conclusion, in our opinion what makes our findings attractive is the evidence that sphingolipid metabolism may represent a target for the discovery of novel therapeutic strategies in HD, especially given that drugs working through its related pathways are already in clinical trial for different other pathological conditions^73,74^.

## Electronic supplementary material

Supplementary Info
