# Author Correction: Defective Sphingosine-1-phosphate metabolism is a druggable target in Huntington’s disease

**DOI:** 10.1038/s41598-018-23083-1

**Published:** 2018-05-24

**Authors:** Alba Di Pardo, Enrico Amico, Abdul Basit, Andrea Armirotti, Piyush Joshi, M. Diana Neely, Romina Vuono, Salvatore Castaldo, Anna F. Digilio, Francesco Scalabrì, Giuseppe Pepe, Francesca Elifani, Michele Madonna, Se Kyoo Jeong, Bu-Mahn Park, Maurizio D’Esposito, Aaron B. Bowman, Roger A. Barker, Vittorio Maglione

**Affiliations:** 1 IRCCS Neuromed, Pozzilli, Italy; 2 Department of Drug Discovery and Development, Fondazione Istituto Italiano di Tecnologia, Genova, Italy; 3 Departments of Pediatrics, Neurology and Biochemistry, Vanderbilt University (VU) and VU Medical Center Pediatric Neurology Research Lab, Nashville, TN USA; 4 John van Geest Cambridge Centre for Brain Repair, Department of Clinical Neuroscience, University of Cambridge, Cambridge, UK; 5 Institute of Biosciences and Bioresources (IBBR), National Research Council (CNR), Naples, Italy; 6 Department of of Cosmetic Science, Seowon University, Cheongju, Korea; 7 NeoPharm USA Inc. Engelwood Cliffs, New Jersey, USA; 8 Institute of Genetics and Biophysics “A. Buzzati-Traverso”, Naples, Italy

Correction to: *Scientific Reports* 10.1038/s41598-017-05709-y, published online 13 July 2017

The original version of this Article contained a typographical error in the spelling of the author M. Diana Neely, which was incorrectly given as Diana M. Neely. This has now been corrected in the PDF and HTML versions of the Article as well as the Supplementary Information that now accompanies the Article.

