# Supplementary material for: Defective Sphingosine-1-phosphate metabolism is a druggable target in Huntington’s disease
Source: Sci Rep. 2017 Jul 13;7:5280. doi: 10.1038/s41598-017-05709-y (PMC5509685; doi:10.1038/s41598-017-05709-y)
Supplement: Supplementary file 1 — Supplementary Info [file 41598_2017_5709_MOESM1_ESM.pdf]

Supplementary Information

Defective Sphingosine-1-phosphate metabolism is a druggable target in Huntington's disease

Alba Di Pardo[a], Enrico Amico[a], Abdul Basit[b], Andrea Armirotti[b], Piyush Joshi[c], M. Diana Neely[c], Romina Vuono[d], Salvatore Castaldo[a], Anna F Digilio[e], Francesco Scalabrì[a], Giuseppe Pepe[a], Francesca Elifani[a], Michele Madonna[a], Se Kyoo Jeong[f], Bu-Mahn Park[h], Maurizio D'Esposito[a,i], Aaron B Bowman[c], Roger A Barker[d] and Vittorio Maglione[a*].

[a]IRCCS Neuromed, Pozzilli, Italy

[b]Department of Drug Discovery and Development, Fondazione Istituto Italiano di Tecnologia, Genova, Italy

[c]Departments of Pediatrics, Neurology and Biochemistry, Vanderbilt University (VU) and VU Medical Center Pediatric Neurology Research Lab, Nashville, TN, USA

[d]John van Geest Cambridge Centre for Brain Repair, Department of Clinical Neuroscience, University of Cambridge, Cambridge, UK

[e]Institute of Biosciences and Bioresources (IBBR), National Research Council (CNR), Naples, Italy

[f]Department of of Cosmetic Science, Seowon University, Cheongju, Korea

[h] NeoPharm USA Inc. Engelwood Cliffs, New Jersey, USA

[i]Institute of Genetics and Biophysics "A. Buzzati-Traverso", Naples, Italy

* Correspondence to: Vittorio Maglione, PhD. Centre for Neurogenetics and Rare Diseases, IRCCS Neuromed, Località Camerelle, 86077 Pozzilli (IS), Italy.

Email: vittorio.maglione@neuromed.it

Supplementary Figures

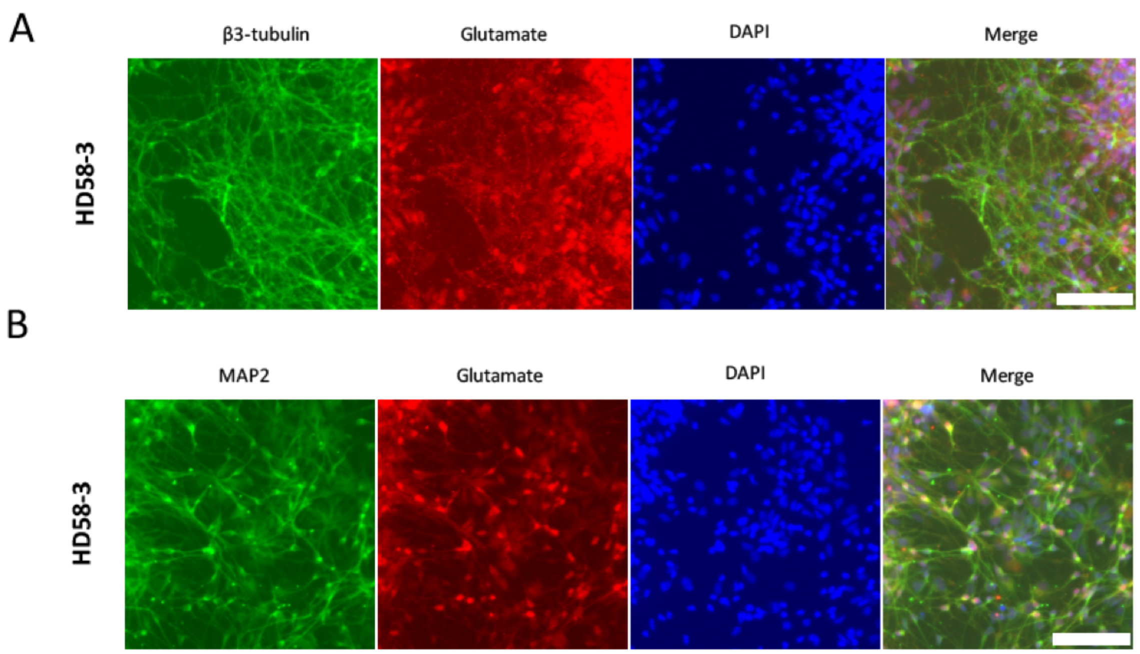

Supplementary Figure 1. **Immunohistochemistry of HD (HD58-3) human iPSC-derived cortical neurons at day 33.** Neurons were stained for β3-tubulin (**A**) and glutamate to confirm generation of glutamatergic neurons and with MAP2 (**B**), a marker of neuronal processes. Scale bars = 100μm.

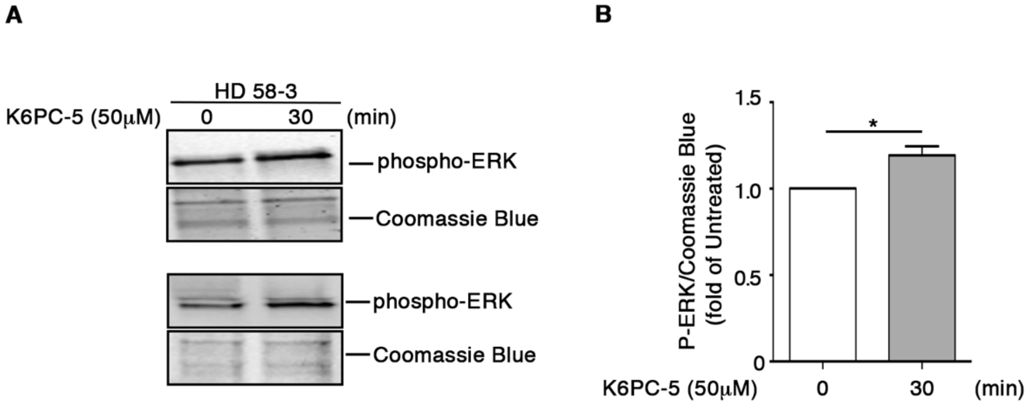

Supplementary Figure 2. Treatment with K6PC-5 increased levels of phospho-ERK in human iPSC-derived human cortical neurons. Cropped immunoblottings (**A**) and densitometric analysis (**B**) of ERK phosphorylation in iPSC-derived HD human neurons after 30 minutes of treatment with 50μM K6PC-5. Coomassie blue staining was used for used for protein normalization. Values are mean ± SD of two experiments. *, $p<0.05$ (Unpaired t-test).

Supplementary Materials and Methods

Human iPSC-derived cortical neurons differentiation stainings. All the staining was carried out after fixation in 100% methanol for 20 min. All the cells were incubated at 4°C with primary antibodies: β3-tubulin 1:500 (Thermo Scientific Cat. N. MA1-19187), MAP2 1:200 (Zymed/Novex Cat. N. 131500), and Hoechst 1:500 (Thermo Fisher Scientific Cat. N. H3570). Appropriate secondary antibodies were added overnight at 4°C in dark (Supplemenatry Fig. 1). Images were taken on a fluorescence microscope, Zeiss Axio Observer Z1 Stand Mot.
